# Climate change and the biodiversity of alpine ponds: Challenges and perspectives

**DOI:** 10.1002/ece3.10883

**Published:** 2024-02-07

**Authors:** Marie Lamouille‐Hébert, Florent Arthaud, Thibault Datry

**Affiliations:** ^1^ INRAE, UR RiverLy, Centre Lyon‐Grenoble Auvergne‐Rhône‐Alpes Villeurbanne Cedex France; ^2^ FNE Haute‐Savoie Pringy France; ^3^ Pole R&D ECLA, OFB, Direction de la Recherche et de l'Appui Scientifique Birieux France; ^4^ Univ. Savoie Mont Blanc, INRAE, CARRTEL Thonon‐les‐Bains France

**Keywords:** connectivity, hydroperiod, species distribution, species richness, water temperature

## Abstract

Inland waters are among the most threatened biodiversity hotspots. Ponds located in alpine areas are experiencing more rapid and dramatic water temperature increases than any other biome. Despite their prevalence, alpine ponds and their biodiversity responses to climate change have been poorly explored, reflecting their small size and difficult access. To understand the effects of climate change on alpine pond biodiversity, we performed a comprehensive literature review for papers published since 1955. Through analysis of their geographic distribution, environmental features, and biodiversity values, we identified which environmental factors related to climate change would have direct or indirect effects on alpine pond biodiversity. We then synthesized this information to produce a conceptual model of the effects of climate change on alpine pond biodiversity. Increased water temperature, reduced hydroperiod, and loss of connectivity between alpine ponds were the main drivers of biodiversity geographic distribution, leading to predictable changes in spatial patterns of biodiversity. We identified three major research gaps that, if addressed, can guide conservation and restoration strategies for alpine ponds biodiversity in an uncertain future.

## Graphical abstract

To understand the effects of climate change on alpine pond biodiversity, we performed a comprehensive literature review for papers published since 1955. Increased water temperature, reduced hydroperiod, and loss of connectivity between alpine ponds were the main drivers of biodiversity geographic distribution, leading to predictable changes in spatial patterns of biodiversity.

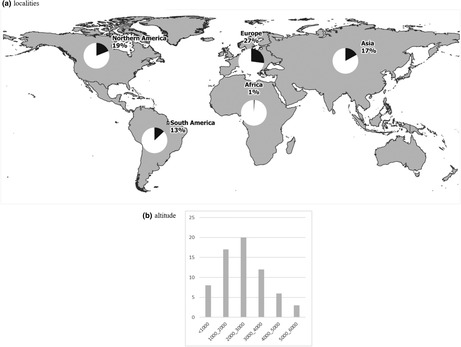

## 1 INTRODUCTION

Inland waters are among the most threatened biodiversity hotspots (Reid et al., 2019; Tickner et al., 2020). In addition to containing ~13% of all described species while representing less than 2% of the globe surface, they provide key ecosystem services to people and society, including provision of drinking water, regulation of climate, and food production (Reid et al., 2019; Tickner et al., 2020). However, climate change threatens the biodiversity of inland waters (Fenoglio et al., 2010; Strayer, 2010; Woodward et al., 2010). Increasing air temperature and the alteration of precipitation patterns increase the frequency and severity of extreme weather events, such as droughts and floods (Barnett et al., 2005; Gobiet et al., 2014; Milly et al., 2005). These changes impose constraints on inland freshwater biodiversity with documented effects on species extinction (Pounds et al., 2006) and geographical distribution (Comte et al., 2013; Hickling et al., 2005, 2006) and phenology (Gibbs & Breisch, 2001; Winder & Schindler, 2004).

Alpine ecosystems, which represent 2.64% of land outside Antarctica (Testolin et al., 2020), experience more rapid climate effects than any other biome (Beniston, 2005; Duan & Xiao, 2015; Pepin et al., 2015). Moreover, alpine areas constitute highly diverse terrestrial and aquatic habitats (Haslett, 1997), with high degrees of endemism (Dirnböck et al., 2011; Körner, 2004; Steinbauer et al., 2016). Alpine areas are thus sentinels of the on‐going and future effects of climate change on biodiversity (Hauer et al., 1997; Williamson et al., 2009). For example, while the global increase in air temperature is 0.25°C/decade (O'Reilly et al., 2015), the European Alps (1000–2700 m asl) experienced an increase in 0.36°C/decade (Gobiet et al., 2014; Thuiller et al., 2005). This alpine temperature effect is compounded by changes in precipitation, radiation, and relative humidity that have led to decreases in snow cover, glaciers volume and extent, and increased drying of freshwater habitats (Chatterjee et al., 2010; Gobiet et al., 2014; Paillex et al., 2020). These changes have measurable effects on the biodiversity of alpine ecosystems.

Alpine biodiversity is already constrained by high‐altitude extreme conditions, such as radiation exposure and high winds. For example, many alpine species require pigmentation or aquatic lifestyles to protect them from high‐altitude UV‐B radiation, which increases by 19% per 1000 m of elevation (Blumthaler et al., 1992; Redmond, 2018). Likewise, flying species need to generate the required power and wing size to adapt to approximately 5% increase of airspeed per 1000 m increase in altitude and low air density (Hedenström et al., 2002).

The effects of climate change on alpine biodiversity are well‐documented across ecosystem types, including terrestrial (Dirnböck et al., 2011; Hoffmann et al., 2019; Weil et al., 2021) and aquatic (Bruno et al., 2019; Green et al., 2022; Niedrist & Füreder, 2023). For example, glacier‐runoff reduction can lead to an increase of algal and herbivore biomass in streams (Cauvy‐Fraunié et al., 2016). In addition, after the loss of hydrological connectivity to a glacier, stopping the influx of “glacier dust,” turbid lakes can turn clear with subsequent effects on community structure due to increased ultraviolet radiation (Kammerlander et al., 2016; Tiberti et al., 2020).

Alpine ponds are one of the most biodiverse alpine ecosystem, hosting endangered and endemic species around the world (Khan & Baig, 2017; Yang et al., 2017). Still, the fate of alpine ponds and their biota is uncertain under climate change, as melting glaciers may reveal new ephemeral habitats at high altitudes, while simultaneously, habitats at lower altitudes may dry up due to reduced hydroperiod (Diaz et al., 2020; Matthews & Vater, 2015; Salerno et al., 2014). Specifically, ponds, natural, or man‐made are defined as being <5 ha in surface area with a water depth of <5 m, and can exhibit permanent or intermittent hydroperiods (Biggs et al., 2005; Richardson et al., 2022). Thus, due to their small size and limited accessibility, alpine ponds and their biodiversity response to climate change remain a major research gap (Khan & Baig, 2017).

In contrast to alpine ponds, the biodiversity response of lowland ponds to climate change is well‐studied (Biggs et al., 2005; Herstoff & Urban, 2014; Thompson & Shurin, 2012), providing an important theoretical framework for predicting effects at higher altitudes. For example, it is predicted that alpine pond biodiversity is at high risk to climate change due to the high sensitivity of their species to temperature increases, hydroperiod alteration, and the gradual isolation of alpine ponds (Carlson et al., 2020). These general predictions still need to be tested and synthesized; however, as species are unequally vulnerable to climate change and because alpine ponds have unique temperature and hydrologic regimes.

Climate change will not affect all species equally, challenging our ability to predict overall biodiversity responses across alpine ponds. Indeed, certain species may even be favored under climate change due to the disappearance of ice barriers and competitors, or perhaps due to preferable changes in food webs (Cauvy‐Fraunié et al., 2016; Redmond, 2018; Seimon et al., 2007). Broadly however, warmer temperatures lead to expected changes in species phenology (e.g., early emergence), size and fecundity (Harper & Peckarsky, 2006), and geographical distribution (Lindholm et al., 2015; Pallarés et al., 2020). Furthermore, water temperature increase is both a direct (via thermal sensitivity) and indirect (e.g., via hypoxia) physiological stress on many aquatic species (Diamond et al., 2023; Pörtner, 2001). These water temperature effects are exacerbated by reductions in water availability due to drying (Diamond et al., 2023).

Annual hydroperiod—the timing and the length of time that there is standing water at a location (Convertino et al., 2013)—is among the strongest filters for species persistence in alpine ponds (Ryan et al., 2014), and in ponds more generally (Wellborn et al., 1996). Hydroperiod decrease due to drying can affect species' size, phenology (Denoël, 2003; Galatowitsch & McIntosh, 2016), and geographical distribution (Sandvik & Odland, 2014; Thurman & Garcia, 2019). In addition to shrinking ecosystem surface area, drying reduces water volume and thermal inertia so that daily water temperature in shallow alpine ponds can fluctuate up to 30°C (Lund et al., 2016; Wissinger et al., 2016). These changes propagate to organismal interactions, especially in the differing responses of resident versus range‐shifting species (Shepard et al., 2022). The gradual geographic isolation of suitable habitat under drying increases the distance among alpine ponds (Ashrafzadeh et al., 2019; Simaika & Samways, 2015), reducing their connectivity. As possibilities for alpine pond species adaptation to climate change are limited, this loss of connectivity reduces the accessibility of suitable niches. Some species may thus shift their ranges to higher elevation (Baur & Baur, 2013; Kelly & Goulden, 2008; Parmesan & Yohe, 2003), but if those ranges exceed peak altitudes, there is an absence of suitable habitat.

In this review, we explored the main drivers and effects of climate change on the biodiversity of alpine ponds. We first conducted a systematic review of the literature to address: (1) the geographic distribution of studied alpine ponds, (2) their biodiversity, and (3) environmental controls on this biodiversity. We then synthesized this information to develop a conceptual model of climate change effects on alpine pond biodiversity. Finally, we used this model to identify research gaps and possible mitigation actions to protect alpine ponds prone to an uncertain future.

## 2 METHODS

We performed a comprehensive literature review using the search engine in Clarivate Web of Science (WoS; Core Collection) for papers published between January 1, 1955, and December 14, 2021. We created a research query including at least the terms “biodiversity” and “climate change”. The query also included two word groups associated with our specific topic of interest: (1) topography (“mountain”, “alpine”, “high‐elevation”), (2) and pond habitat (“ponds”, “waterbody(ies)”, “standing water”, “tarns”, “kettleholes”, “wetland”, “bog”, “peatland”, “fen”, “bofedale(s)”). This search led to 316 results, all of which were written since 1997, with 48% of them written after 2018. From these papers, we found that 73 of those referred to ponds' biodiversity and climate change within alpine regions.

We organized the information from each paper in a database (see Appendix S1) according to the following: (1) study location (e.g., country, altitude), (2) study biological group (e.g., macroinvertebrates, flora), (3) “essential biodiversity description variables” (EBV) defined as *biological state variables that are measurable at particular points in time and space to document biodiversity change* (e.g., community composition) (Pereira et al., 2013; Schmeller et al., 2017), (4) pond typology (e.g., pond, shallow lake, cf. Richardson et al. (2022)), (5) pond geometry (e.g., depth, surface), and (6) environmental drivers of diversity (e.g., water quality, connectivity). We classified environmental factors affecting EBVs into explanatory variable groups (Table 1).

**TABLE 1:** Different explanatory variables are used to study the effects of climate change on the biodiversity of alpine ponds. They are grouped into 12 categories.

Topography	Controlling catchment type, snow coverage, and lakes connectivity
Water quantity	Hydrodynamics (type and volume of inflow/outflow), drought/hydroperiod, water‐level/depth fluctuations (or not fluctuation), evaporation, glacier influence
Temperature	Latitude, longitude, altitude, air temperature, water temperature
Alpine ponds' characteristics	Pond surface area, pond depth, catchment size
Aquatic habitat	Aquatic vegetation, microhabitat, freshwater habitat structure, absence/presence of sediment, pond successional stage
Land cover	NDVI, environmental heterogeneity
Water quality	Trophy, PC, nutrient concentration, catchment lithology, water browning, transparency, turbidity
UV radiation	
Pollutions	Atmospheric and organic pollutants
Connectivity	Hydrologic and topographic
Biotic pressure	Competition/biotic interaction, fish, biological invasions, parasitism
Anthropic pressure	Fire, pasturage, land use, human disturbance, grazing

Biodiversity is variously quantified across studies (e.g., functional versus genetic, alpha versus beta), complicating synthesis efforts. To simplify interpretation, we chose to aggregate biodiversity measures in accordance with the six EBV classes (Pereira et al., 2013) as follows:

1. genetic composition (e.g., allelic diversity),
2. species traits (e.g., phenology),
3. ecosystem function (e.g., nutrient retention),
4. ecosystem structure (e.g., ecosystem vertical profile),
5. species populations (e.g., abundances), and
6. community composition (e.g., taxonomic diversity).

## 3 RESULTS

### 3.1 The geographic distribution of published studies on alpine ponds

**FIGURE 1:**
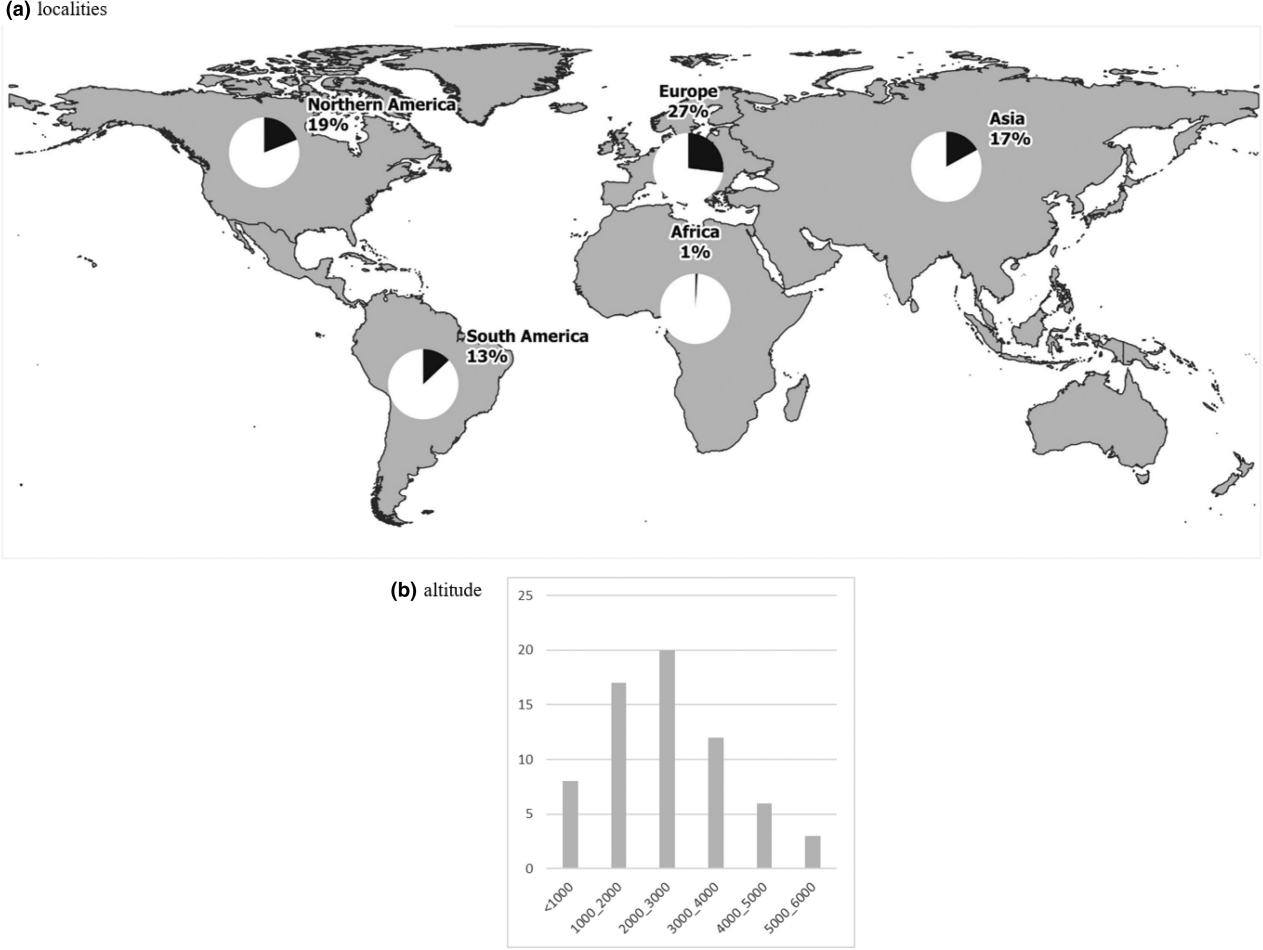
Alpine ponds papers distribution (a: localities, *N* = 73; b: altitude, *N* = 66).

Alpine ponds were predominately investigated in America and Europe (respectively 32% and 38% of the studies; Figure 1a), with the Rocky Mountains, the Andes, and the Alps being the most studied mountain ranges. This is a heavily skewed sampling distribution as Europe and America represent only 2% and 24% of non‐polar alpine areas, respectively (Testolin et al., 2020). Indeed, Asia represented only 17% of studies despite constituting 73% of all alpine area (Testolin et al., 2020), largely concentrated in the Himalaya/Qinghai‐Tibetean mountainous region (Chatterjee et al., 2010; Gujja et al., 2003). Africa comprises the smallest alpine area (<1% (Testolin et al., 2020)) but was home to 6% of alpine pond studies. There were no alpine pond studies from Oceania, but one study in Tasmania used *Chironomidae* spp. diversity to infer post‐glacial and Holocene paleoclimates (Rees & Cwynar, 2010).

The majority (74%) of studies occurred between 1000 and 4000 m asl (Figure 1b). Of the papers that included lower‐altitude ponds under 1000 m asl (the mean tree line elevation) in their study design (12%), most were used as endmembers of community responses to an altitudinal gradient. High‐altitude alpine ponds between 4000 and 6000 m asl were less studied (14%) and were exclusively in Himalaya/Qinghai‐Tibetean and Andean mountains.

### 3.2 The biodiversity of alpine ponds

Alpine pond species richness decreased with increasing altitude (Oertli, 2010), as a function of increasingly constrained drivers of temperature (Oertli, 2010), hydroperiod (He et al., 2016; Sandvik & Odland, 2014; Thurman & Garcia, 2019) and geographical connectivity (Hill et al., 2021; Oertli et al., 2008). Geographical connectivity of alpine ponds may be defined as the Euclidean distance from one pond to another, allowing direct comparison with species' dispersal distances, or the presence of tributaries connecting one pond to another. Whereas forests and topographic relief reduce dispersal distances of alpine species, rivers, lakes, and wetlands can act as corridors, enhancing geographical connectivity (Engler et al., 2009). Indeed, deeper water bodies like lakes act as potential population sources for alpine ponds and are habitat substitutes when alpine ponds are dried (Bouvier et al., 2009; Hortal et al., 2014; Ilg & Oertli, 2014).

#### 3.2.1 Indicator groups to investigate the effects of climate change on alpine pond biodiversity

**FIGURE 2:**
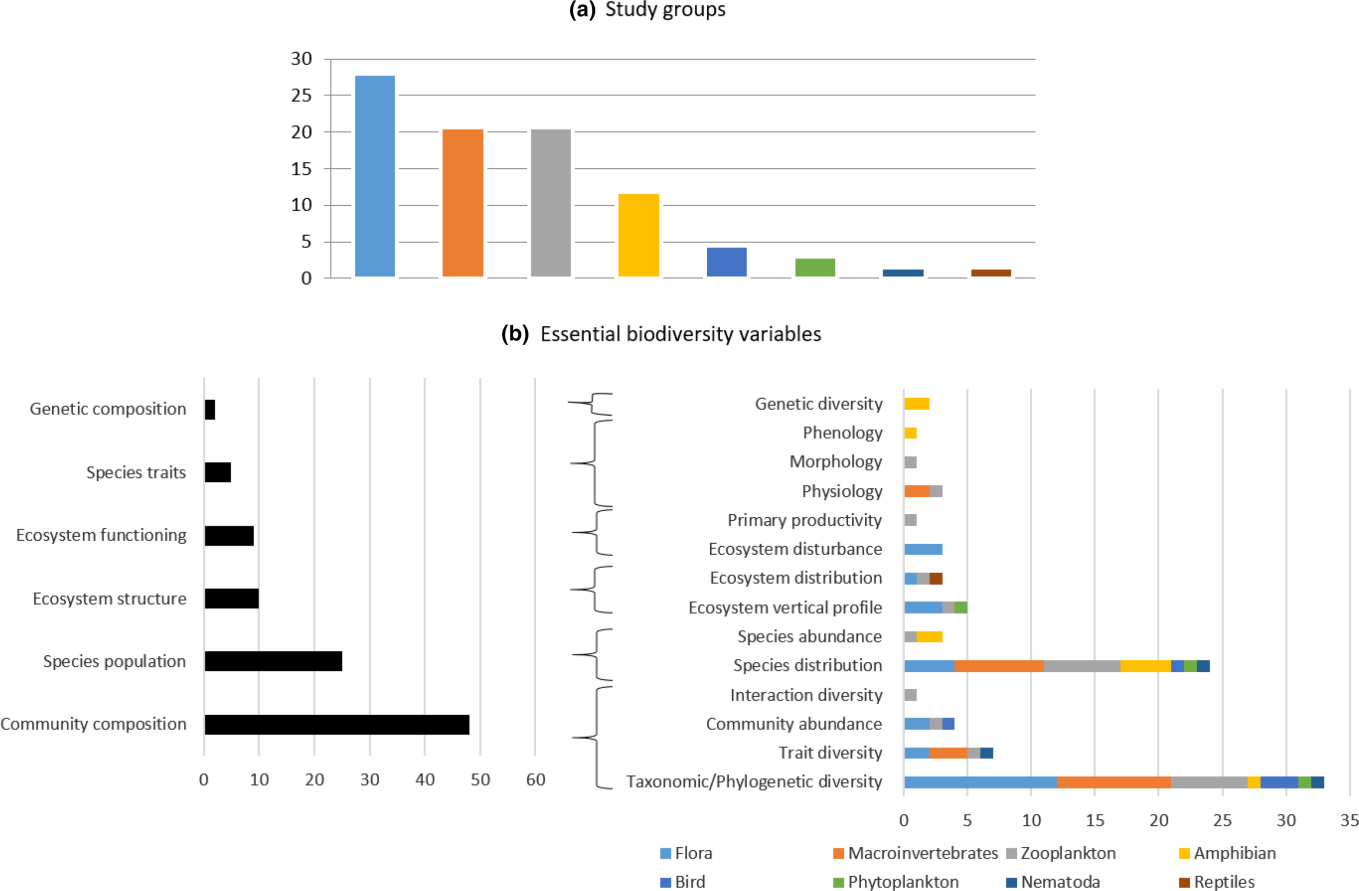
Different (a) study groups and (b) essential biodiversity variables (i.e., EBV, measurement required for study, reporting, and management of biodiversity change (Pereira et al., 2013)) used to study the effect of climate change on alpine ponds' biodiversity. « Community composition » (48) and « Species population » (25) are the principals EBV' applied on the 73 reviewed papers. They are numerous because of the large share of articles respectively on « Taxonomic/Phylogenetic diversity » (31) and « Species distribution » (22). Most of the EBVs have been studied for some faunal or floral groups (1–4) with the exception of « Taxonomic/Phylogenetic diversity» and « Species distribution », which concern seven groups.

The effects of climate change on alpine pond biodiversity were primarily studied on species for which all or part of their life stages are strictly aquatic. The distribution of biodiversity studies according to guild was as follows: flora (including phytoplankton) (31%), macroinvertebrate (22%), zooplankton (21%), and amphibians (12%) (Figure 2a). These groups, including numerous endangered species, appeared as highly relevant climate change indicators because their life history strategies (distribution, composition, and phenology), behavior, and physiology makes them particularly sensitive to climate change.

#### 3.2.2 Controls on alpine pond biodiversity responses to climate change

Each of the six EBVs were used to study the effect of climate change on alpine pond biodiversity (Figure 2b).

1. Genetic composition: Only two papers considered genetic composition in alpine ponds, both on amphibian phylogeography (Ben Hassine et al., 2016; Gonçalves et al., 2015). They focused on the effect of climate change on alpine pond population persistence, differentiation, and incipient speciation.
2. Species traits: Only five papers studied species traits in alpine ponds. Phenology, physiology and morphology were studied across macroinvertebrates, zooplankton, and amphibians to understand resistance to temperature extremes (Hotaling et al., 2021; Lindholm et al., 2015; Pallarés et al., 2020) and to predatory fish invasions (Denoël et al., 2019). For example, increasing water temperature modified the physiological traits (e.g., growth and respiration) of the Arctic fairy shrimp (*Branchineta paludosa*) up to a threshold temperature, at which point it disappeared from the pressured pond (Lindholm et al., 2015).
3. Ecosystem function: Ecosystem disturbance in alpine ponds linked with climate change was investigated only on flora in pond–peatland complexes (Chatanga & Seleteng‐Kose, 2021; Hämmerle et al., 2018; Volkova et al., 2021), and primary productivity only on alpine pond phytoplankton (Thompson et al., 2008). Warming combined with other drivers (e.g., nitrogen deposition, land use, and pollution) led to changes of the composition of alpine pond communities (Thompson et al., 2008; Volkova et al., 2021). These changes can for example modify the community capacities of assimilation of nitrates and dissolved gaseous nitrogen (Thompson et al., 2008).
4. Ecosystem structure: Paleoecological indicators (e.g., pollen, flora and fauna fossils) are used in sediment cores to study the long‐term evolution of community composition of alpine peatlands with open‐water pools (Connor et al., 2018; Rodríguez & Behling, 2011). These studies describe the changing effects (since the late Holocene) of climate and land‐use pressure (e.g., fire, farming, and upper forest line) on pond biodiversity.
5. Species populations: Alpine pond species geographical distribution were studied (27% of selected papers) to understand how they react to climate change effects. Species move with the variation of their suitable habitat distribution, in relation to increase of temperature (Pallarés et al., 2020), frequency and duration of heatwaves (Carlson et al., 2020), glacier retreat (Seimon et al., 2007) and competition with valley and/or parasites species (Lindholm et al., 2016). For cold specialist species, warming and drying led to adaptation (Pallarés et al., 2020), or regional scale retraction of their distribution area (with local extinction) (Carlson et al., 2020; Lindholm et al., 2016). In addition, alpine ponds were colonized by warmer tolerant species that were able to extend their range (Seimon et al., 2007). These species can be potential competitors for alpine pond specialist species making their joint survival more difficult (Shepard et al., 2022).
6. Community composition: Studies were done on alpine ponds using principally taxonomic/phylogenetic diversity (42% of selected papers). Warming led to broad changes on species richness (Redmond, 2018; Sandvik & Odland, 2014). For example, no specialist species colonize alpine ponds when rare specialist species are disappearing (Sandvik & Odland, 2014). But also, communities can shift to smaller body size species more adapted to warming conditions (Redmond, 2018).

Overall, species distributions and taxonomic/phylogenetic diversity best described the effects of rapid climate change on alpine pond biodiversity. Indeed, temperature increase at high altitudes is so rapid that in situ possibilities of species adaptation are limited (Baur & Baur, 2013; Pallarés et al., 2020; Parmesan, 2006). Using principally these two EBVs at the scale of populations and communities, we can describe how other environmental factors (e.g., water quality) influence the biodiversity of alpine ponds.

### 3.3 Environmental controls shaping the biodiversity of alpine ponds

To better predict the effects of climate change on alpine pond taxonomic/phylogenetic diversity and species distribution, we identified the three dominant environmental controls (Table 1) common to the 73 selected studies. The main drivers studied on taxonomic/phylogenetic diversity were temperature (*n* = 21), water quantity (*n* = 17), water quality (*n* = 12), land cover (*n* = 9), connectivity (*n* = 9) and biotic pressure (*n* = 8). On species distribution they were temperature (*n* = 17), biotic pressure (*n* = 9), water quantity (*n* = 8), and connectivity (*n* = 7). For these two EBVs, temperature and water quantity were the a priori most constraining environmental controls (and the most studied), with connectivity following close behind. To understand the effects of climate change on alpine pond biodiversity, we explored the effects of these three main drivers: temperature, hydroperiod (for water quantity), and connectivity (hydrologic and topographic).

#### 3.3.1 Temperature

Alpine ponds are post‐glacial refugia (Pallarés et al., 2020) for cold specialist/stenotherm species that have undergone a long process of adaptation to environmental constraints. For example, due to cold water and air temperatures, species exhibit a short activity (growth) period when alpine ponds are free of ice and snow (Céréghino et al., 2012; Hinden et al., 2005; Lindholm et al., 2015). At least 24 species (birds, vertebrates, invertebrates, flora) are endemic to alpine ponds (Chatanga et al., 2019; Izaguirre et al., 2018; Pallarés et al., 2020) because many of them remain isolated in these high‐altitude areas (Sharma et al., 2015). Still, the total number of endemic alpine pond species is unknown and it is thought that they are among the most endangered in the world (Rosset & Oertli, 2011).

Alpine pond species distributions are limited by air temperature. For macroinvertebrates and amphibians, cold thermal specialist species have been described in Switzerland located at sites with a mean annual air temperature not exceeding 6°C (Rosset & Oertli, 2011). Although they are able to endure large temperature ranges over daily timescales (e.g., >10°C in Norway), these species are sensitive to high temperatures (Epele et al., 2022; Lindholm et al., 2015) and are geographically restricted to a small thermal range (Rosset & Oertli, 2011). Experimental studies of beetles (*Agabus nevadensis* and *Hydroporus sabaudus sierranevadensis*) in Spain demonstrated that while increased air temperature was not lethal to adults, long exposure (>10 days) to warmer temperatures induced oxidative stress (Pallarés et al., 2020). Still, it is unclear how warming may affect less tolerant larvae.

Water temperature determines alpine ponds' community composition (Miller et al., 2021). Warmer waters have interactive effects with predation patterns on species growth, distribution, and intra‐guild competition (Loewen et al., 2020; Owens et al., 2023; Shepard et al., 2022; Symons & Shurin, 2016). For example, as waters warm and species migrate to higher elevations, preferential consumption of large body zooplankton by predators (*Oncorhyncus mykiss*) allows smaller body zooplankton to dominate colonization patterns (Jones et al., 2020). Moreover, increased water temperature preferentially reduces larger zooplankton populations due to their temperature‐sensitive metabolism, leaving smaller, more metabolically plastic zooplankton species to fill their niche (Redmond, 2018). Increased metabolic and energetic demand induced by temperature increase can also reduce total animal biomass (Bruno et al., 2019), and subsequently less available dispersal energy. Indeed, many cold water specialists that were present at the beginning of the 20th century are now absent in alpine ponds, following trends from boreal and arctic regions (Iglikowska & Namiotko, 2012).

By contrast, thermal generalist species occupy a wider distribution area than specialists and can more rapidly colonize the shifting thermal landscape (Rosset & Oertli, 2011; Sturm, 2012). If connectivity is established, generalist species can colonize higher and cooler sites as lower sites begin to warm. For example, anurans and their pathogens (*Chytridiomycosis* spp.) were able to move 156 m higher (5244 to 5400 m) in 10 years (Seimon et al., 2007). In addition to their mobility, some generalist species can adapt to cooler temperatures: For example, decreasing their body size and consequently their need for nutrients or periodically going out of the water. They are characterized by higher abilities to colonize new habitats when the other species of the same taxonomic group are not (Sturm, 2012).

As air temperature increases, increasingly more generalist species are arriving from lower altitudes with warmer and drier conditions when specialist species are decreasing (He et al., 2016; Stiles et al., 2017). For example, alpine pond bryophytes are being replaced by vascular plants (He et al., 2016), and birds from lower altitude are now established on the Bogota Region in Colombia (Stiles et al., 2017). This overall upward trajectory shrinks the range for native alpine pond species, like the Arctic fairy shrimp (*Branchinecta paludosa*), whose occurrence was reduced over 41 years from 77 ponds to 59 ponds in a pool of 121 ponds (Lindholm et al., 2012), due to its maximum thermal tolerance of 12.7°C (Lindholm et al., 2015).

Although increased temperatures are driving specialist species' disappearance, replacement generalists are likely causing alpine ponds to experience a simultaneous increase in overall taxonomic/phylogenetic diversity and a homogenization (Iglikowska & Namiotko, 2012). Still, few studies have documented direct links with increased temperature and alpine pond biodiversity, and we found no work on increased temperature effects on alpine pond snow cover or freezing. Nevertheless, we hypothesize that ice or snow cover period shortening will reduce alpine pond biodiversity linked for example to phenological prey–predator desynchronization.

#### 3.3.2 Hydroperiod of alpine ponds

Although the effects of hydroperiod have been little studied in alpine pond biodiversity, the few studies that have been done converge on similar results obtained in many studies done in freshwater depressional wetlands of lower altitude (Boix & Batzer, 2016; Epele et al., 2022; Kneitel, 2014). Hydroperiod decrease can lead to modification of community composition, species traits and distribution, or to species disappearance. For example, mesocosm experiments linked lowered hydroperiod to reduced biomass of amphibian individuals at emergence (Thurman & Garcia, 2019). Reduced hydroperiod can also cause community composition modification and decrease of species distributions. In Norway alpine ponds, between 1979 and 2010, hygrophilic plants (e.g., *Eriophorum angustifolium*, *Carex nigra*) occurrence decreased, coinciding with colonization by species of drier environments, like graminoids (*Calamagrostis neglecta*, *Deschampsia alpina*) (Sandvik & Odland, 2014). Likewise, some bryophytes are now absent in areas that have undergone hydroperiod reduction (He et al., 2016). Moreover, the 2017 summer heat waves dried out alpine ponds in Chamonix, which led to *Rana temporaria* tadpoles mortality (Carlson et al., 2020).

Some cold specialist species can be adapted to hydroperiod variability. Like *Somatochlora alpestris*, some boreo‐alpine odonata species have been seen burrowing in the peat to resist drought effects (Grand & Boudot, 2006; Heidemann & Seidenbusch, 2002; Wildermuth, 2013). However, these resistances were not measured (e.g., duration of resistance, soil moisture threshold). Some amphibians may adopt other resistance strategies, such as plasticity in metamorphosis timing as a function of hydroperiod conditions (Denoël, 2003). Indeed, phenological plasticity appears to be the most common resistance strategy to hydroperiod reduction across generalist (Izaguirre et al., 2018; Laurila et al., 2002) and specialist species, alike (Laurila et al., 2002; Nylin & Gotthard, 1998). Still, species are ultimately limited by timing and duration of pond drying, and few species are adaptable to entire gradient of hydroperiod reduction (Galatowitsch & McIntosh, 2016).

Climate change is leading to variation of temperature and precipitation patterns (Barnett et al., 2005; Gobiet et al., 2014; Milly et al., 2005). Permanent alpine ponds are becoming temporary, and some temporary are becoming more permanent (Epele et al., 2022; Robinson & Oertli, 2009; Wissinger et al., 2016). The presence of standing water is also controlled by winter ice grip when the entire water volume freezes in shallow systems. Date and duration of water availability are one of the main factors controlling alpine biological cycles (Ryan et al., 2014). Under limited mobility, alpine pond species need to resist in situ to hydroperiod range changes by modifying, for example, their desiccation resistance. In addition, they are undergoing modifications in the composition of the communities, among other things by the arrival of new, more tolerant species. If they cannot adapt rapidly, they need to move to find an alpine pond connected with suitable hydroperiod conditions.

#### 3.3.3 Connectivity

The spatial distribution of alpine ponds is heterogeneous and fragmented, highlighting the importance of regional connectivity in driving biodiversity patterns. Indeed, depending on location within the landscape and connectivity to other suitable habitats, either local or regional processes may drive an individual alpine pond biodiversity (Leibold et al., 2004). For example, macroinvertebrate diversity in Switzerland (Macun Cirque) is positively correlated with regional alpine pond connectivity, but is uncorrelated with local variables (surface area, average water depth, hydroperiod) (Hill et al., 2021; Oertli et al., 2008). Connectivity among alpine ponds is therefore an important component of their metacommunity framework (sensu Leibold et al., 2004). To survive under climate change, alpine pond species need to adapt or to move to a suitable connected habitat. Those that cannot are threatened with extinction.

Alpine pond networks are made up of habitats that rapidly fluctuate in different gradients of hydroperiod. As temperature increase, early melting of snow and glaciers shifts the timing of spring water availability and typically results in a summer hydroperiod decrease and drying of smaller ponds (Robinson & Oertli, 2009). Moreover, glacial margin expansion under warming is leading to a transformation of alpine pond landscapes and networks, like the appearance of ecological corridors, and disappearance/creation of ponds, themselves (Seimon et al., 2007). New connectivity is allowing certain species to colonize new environments, although wind speed and directionality has an important role too for the dispersal of non‐flying terrestrial and aquatic species in alpine areas (Crabtree & Ellis, 2010; Epele et al., 2021). Increased connectivity promotes pathogen (*Batrachochytrium dendrobatidis*) colonization which can lead to extinctions of amphibian populations (Seimon et al., 2007) and subsequent colonization by new species on vacated habitat patches (Leibold et al., 2004; MacArthur & Wilson, 2001). By contrast, habitat isolation under reduced connectivity limits aquatic plant colonization (Arthaud et al., 2013).

Broadly, effects of climate change on biodiversity of alpine ponds is poorly explored compared to alpine terrestrial ecosystems. Indeed, the effect of climate change on habitat connectivity of alpine terrestrial species has been extensively studied (Breshears et al., 2008; Kelly & Goulden, 2008; Parmesan & Yohe, 2003). Alpine pond species appear to follow the same distributional changes related to climate change as terrestrial species. The geographic ranges of many alpine species are changing by contracting, expanding or shifting in the direction of higher altitudes (Breshears et al., 2008; Kelly & Goulden, 2008; Parmesan & Yohe, 2003).

### 3.4 Conceptual model of the effect of climate change on alpine ponds' biodiversity

**FIGURE 3:**
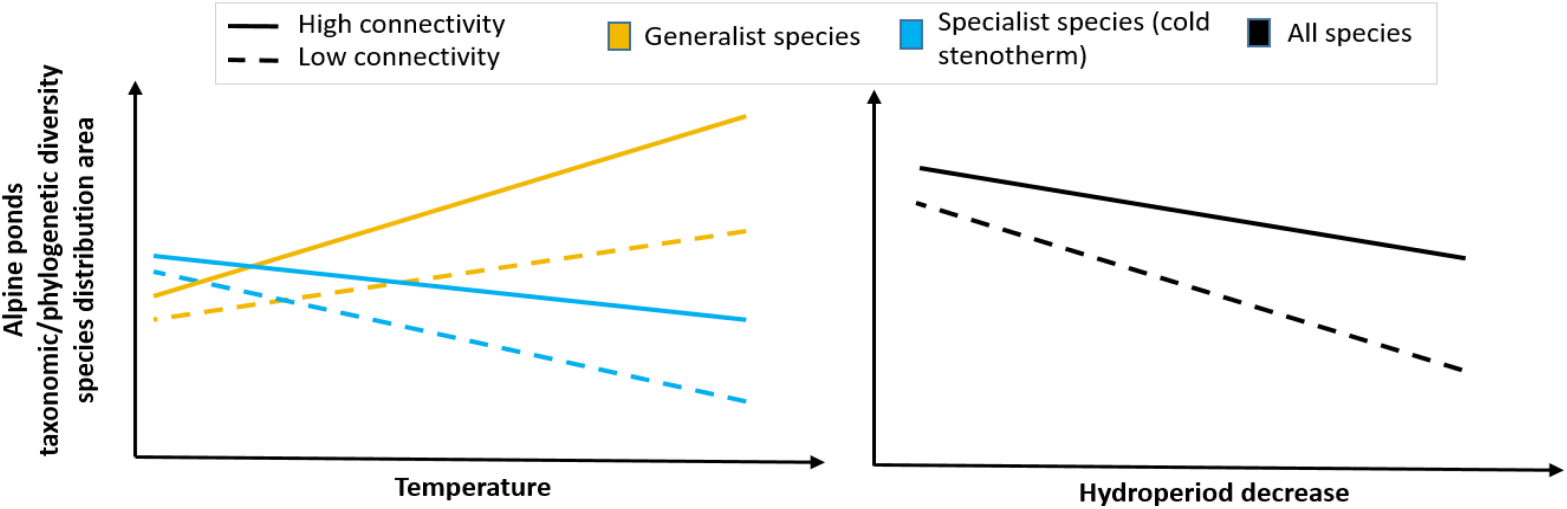
Effects of climate change (temperature, hydroperiod) on alpine ponds' taxonomic/phylogenetic diversity and species distribution area. Warming leads to an increase of alpine ponds' taxonomic/phylogenetic diversity and species distribution area for generalist species (orange), especially since connectivity is high (decrease for specialist species had a higher decrease when alpine ponds are low connected [blue]). Decrease in hydroperiod leads to decrease (stronger when they are poorly connected) of taxonomic/phylogenetic diversity and species distribution area for all species (black).

Our comprehensive review indicated that water temperature, hydroperiod and connectivity are the main drivers of the biodiversity of alpine ponds in the perspective of climate change. We used these three drivers to develop a conceptual model to predict the effects of climate change on the biodiversity of alpine ponds (Figure 3).

From this model, we draw several predictions of the effects of climate change on alpine pond biodiversity. The first prediction is that temperature increase and its associated effect on hydroperiod reduction will shrink cold specialist species distributions and overall taxonomic diversity. The second prediction is that the more connected alpine ponds are, the more the decline of specialist species can be mitigated. In contrast, we predict that generalist species distribution area and taxonomic diversity will be favored by temperature increase. Nevertheless, the decrease in hydroperiod will have similar effects on generalist species as it does on specialist species.

### 3.5 Research gaps

We identified several research gaps related to the three main drivers of the biodiversity of alpine ponds in the perspective of climate change (Table 2). The largest research gap we identified is the thermal range of alpine pond specialist species, which is needed to assess their capacities to adapt and survive under temperature increases. Filling this gap will make it possible to anticipate and guide the restoration or creation of suitable habitats for threatened species. A second research gap is the ability for generalist species to colonize warmed habitats, as these species increase pressure for specialist survival. We should further quantify species' resistance abilities to drying and freezing, as has recently been done for invertebrates in temporary freshwaters (Strachan et al., 2015; Stubbington & Datry, 2013; Williams, 1996). The loss of alpine pond biodiversity under climate change could be attenuated if specialist species are able to colonize novel suitable habitats in higher altitudes or latitudes. Yet, we need to improve knowledge about dispersal capacities of the different stages of alpine pond species. This will allow estimating the thresholds in terms of connectivity among alpine ponds promoting species survival and recolonization. Filling these critical research gaps will allow accurate prediction of current and future species distributions and can help guide conservation strategies for alpine ponds prone to an uncertain future.

**TABLE 2:** Few studies have been carried out to describe and quantify the effects of climate change induced variability of water temperature, hydroperiod, connectivity, on alpine pond biodiversity. Here we list the main research questions we need to develop in order to identify an operational framework for the preservation of specialist alpine pond species threatened by climate change.

Driver	Research questions
Water temperature	What are the cold stenotherm alpine pond species and their thermal ranges?
	Will these ranges be available with climate change?
	What are the physiological resistance pathways to resist temperature increase, amplitude and freezing?
	Which species will benefit from the increase in water temperature to colonize alpine ponds?
	How cold stenotherm species will survive with new species colonizations?
Hydroperiod	What is the duration cold stenotherm alpine pond species could resist drying in summer and freezing in winter?
	Does frequency of drying have significant effects on organism survival?
	What are the effects of intra‐annual drying events (frequencies and duration) on species' distribution?
	What are the physiological resistance pathways for organisms to resist freezing and drying?
	What frequency and duration of drying/freezing is tolerable for the organisms colonizing alpine ponds?
	With early melting of ice and snow on alpine ponds, does predator–prey phenology still synchronize?
Connectivity	What are dispersal abilities of different stages of cold stenotherm alpine pond species?
	What is habitat availability for alpine pond species?
	How land‐use changes (drained ponds, creation of artificial ponds) are already affecting habitat availability for alpine pond species?
	Are connectivity's thresholds ensuring the maintenance of cold stenotherm alpine pond species?
	Can we prioritize connectivity maintenance and restoration between alpine ponds to preserve cold stenotherm alpine pond species?
	What are the dispersal abilities of “winning” species?
	How connectivity maintenance, restoration, and new connectivity (glacier retreat for example) between alpine ponds could allow “winning” species colonization?
	Can we find a way to preserve or to create cold stenotherm species connectivity between alpine ponds without favoring “winning” species colonization?

## AUTHOR CONTRIBUTIONS

**Marie Lamouille‐Hébert:** Conceptualization (equal); data curation (lead); formal analysis (lead); funding acquisition (lead); investigation (equal); methodology (equal); project administration (lead); validation (equal); visualization (equal); writing – original draft (lead); writing – review and editing (lead). **Florent Arthaud:** Conceptualization (equal); investigation (equal); methodology (equal); supervision (supporting); validation (equal); visualization (equal); writing – original draft (supporting); writing – review and editing (supporting). **Thibault Datry:** Conceptualization (equal); investigation (equal); methodology (equal); supervision (lead); validation (equal); visualization (equal); writing – original draft (supporting); writing – review and editing (supporting).

## CONFLICT OF INTEREST STATEMENT

The authors have no conflict of interest with the contents in this manuscript, which are solely the opinions of the authors.

## Supporting information


Appendix S1.

## Data Availability

The authors confirm that they don't have any data.
